# The rise of the distributions: why non-normality is important for understanding the transcriptome and beyond

**DOI:** 10.1007/s12551-018-0494-4

**Published:** 2019-01-07

**Authors:** Jessica C. Mar

**Affiliations:** Australian Institute for Bioengineering and Nanotechnology, University of Queensland, QLD, Brisbane, 4072 Australia

**Keywords:** Non-normality, Gene expression variability, Skewness, Gene expression, Big data, Single-cell sequencing

## Abstract

The application of statistics has been instrumental in clarifying our understanding of the genome. While insights have been derived for almost all levels of genome function, most importantly, statistics has had the greatest impact on improving our knowledge of transcriptional regulation. But the drive to extract the most meaningful inferences from big data can often force us to overlook the fundamental role that statistics plays, and specifically, the basic assumptions that we make about big data. Normality is a statistical property that is often swept up into an assumption that we may or may not be consciously aware of making. This review highlights the inherent value of non-normal distributions to big data analysis by discussing use cases of non-normality that focus on gene expression data. Collectively, these examples help to motivate the premise of why at this stage, now more than ever, non-normality is important for learning about gene regulation, transcriptomics, and more.

## Big data continues to get bigger

Statistics has helped us arrive at many major genomic discoveries, and the uptake of routine statistical and computational methods has been formalized into its own field, namely bioinformatics and computational biology (Gentleman et al. 2004; Stein 2002). Applications that stem from pre-processing of gene expression data up to higher order analyses have collectively contributed knowledge on the dynamic signatures and regulatory rules that define cellular phenotypes (Lockhart et al. 1996; Tamayo et al. 1999; Alon et al. 1999; Schadt et al. 2000). Advances in technology platforms are ushering in an unparalleled expansion of big data where both the size and complexity of datasets are increasing at an accelerated rate (Lowe et al. 2017). This can be seen most readily by the recent confluence of datasets produced by single-cell next-generation sequencing approaches (Liu and Trapnell 2016; Shapiro et al. 2013; Levitin et al. 2018; Oldham and Kreitzer 2018). Limitations to high-throughput data generation are continuing to fall across multiple axes, whether it be through the rapid increase in the number of tissues, genes, cells, or regulatory data types that can be profiled (Koch 2018; Medioni and Besse 2018; Lacar et al. 2016). As big data continues to grow more complex, opportunities for statistical innovation abound. Consequently, there is a pressing need to take stock of the statistical methods being implemented and to determine whether more effective alternatives exist. If we can meet these challenges in a timely and collaborative way, exciting new directions in computational biology await.

## Assumptions make the world go around

**Fig. 1:**
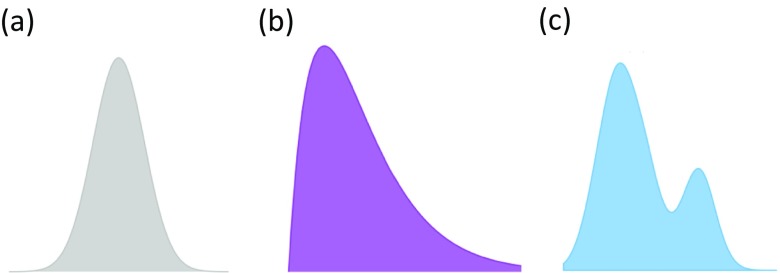
Distributions come in different shapes and sizes. **a** Normal distribution. **b** Gamma distribution. **c** Bimodal distribution

Like any quantitative science, mathematical assumptions are a core tenet of statistics (Casella and Berger 2008; Tukey 1997). Typically, assumptions focus on properties of the data where the most common one is the type of distribution that data follows. As any student of a statistics class will know, the keystone assumption of applied statistics is the normal distribution (Curran-Everett 2017). Normality is a standard assumption that can be worthwhile to make because when it can be applied, powerful artillery of statistical methods can be used. This assumption is not made without a good basis, and in many cases for continuous data, a normal distribution is a reasonable assumption to make. Moreover, the central limit theorem (CLT) (Billingsley 1995), a key result from probability theory, demonstrates that under certain conditions and with asymptotically large amounts of data, sums or averages of data points will approximately follow a normal distribution, even when the data themselves are non-normal. When conditions for the CLT hold, this theorem provides validity to use statistical tools like the *t* test, ANOVA, and linear regression modeling that are familiar and easy to implement. Although the CLT provides a theoretical justification for normality to be assumed, it is worthwhile remembering that in statistics, alternative distributions also exist (Fig. 1).

## Counting reads: how discrete probability distributions have become the standard for modeling transcription from next-generation sequencing methods

For profiling whole transcriptomes, next-generation sequencing methods like RNA-sequencing (RNA-seq) (Mortazavi et al. 2008) have eclipsed the use of microarray-based technologies (Lowe et al. 2017). Because gene expression estimation from RNA-seq methods involve aligning and quantifying the numerous short reads that map back to a reference complementary genome (Wang et al. 2009a), the data output of an RNA-seq experiment is a set of discrete read counts (Conesa et al. 2016). In contrast, microarrays rely principally on the fluorescence of different reference probes to determine the abundance of gene expression and instead the data takes the form of continuous intensities produced by an image scanner (Butte 2002). With the pronounced shift in technology from microarrays to RNA-seq, a corresponding and necessary change in how we model gene expression has also occurred where continuous probability distributions have been replaced by discrete counterparts.

Various families of discrete distributions have been employed for modeling read count data generated by RNA-seq methods. At this stage, most methods converge on the negative binomial. Currently, the standard bioinformatics methods for analyzing RNA-seq read counts share this common feature of using a negative binomial distribution, and examples include DESeq2 (Love et al. 2014), CuffDiff (Trapnell et al. 2010), and edgeR (Robinson et al. 2010; McCarthy et al. 2012). The Poisson distribution is also a natural choice for modeling count data, but a property of this distribution is that the mean and variance are identical. It has been shown that this distributional property is too restrictive for RNA-seq read counts since it is not uncommon for the variance to be larger than the mean gene expression, a phenomenon termed as overdispersion. As a result, the negative binomial distribution has emerged as a more flexible and appropriate option, since under this distribution, the mean and variance are unlinked and modeled by separate parameters. The release of more advanced RNA-seq tools to model read count data continue to feature further improvements, for example, the inclusion of mixed models (Sun et al. 2017; Al Mahi and Begum 2016) or approaches involving Bayesian or empirical Bayes that build upon the use of these discrete probability distributions (Gu et al. 2014; Papastamoulis and Rattray 2018; Leng et al. 2013).

## Seeing double—how bimodal distributions reveal hidden substructures for patient population data

The value that stems from using statistics based on non-normality can be readily seen from the growing number of studies that use bimodal distributions to model RNA expression levels and identify new phenomenon (Liu et al. 2018; Zechner et al. 2012; Karn et al. 2012). One of the most well-known attributes of the normal distribution is the presence of a single mode, which in statistics is defined as the most frequently occurring value in the distribution. However, probability densities can be modeled by a range of distributions, and moreover, depending on the shape of the data, it may be more appropriate to select a distribution that has more than one mode.

Bimodality has been particularly successful for transcriptional profiling datasets from large cohorts of cancer patients because the presence of two subpopulations may indicate new targets of clinical relevance such as markers for tumor subtypes or survival status. For example, using microarray data from epithelial ovarian tumors (Tothill et al. 2008), Kernagis et al. (2012) applied the bimodality index (Wang et al. 2009b) to identify genes with robust bimodal expression profiles and found that these were also differentially expressed between tumor subtypes. The combination of bimodally expressed genes was used to derive a survival score and Kernagis et al. showed that statistically significant differences in patient survival could be determined based on this score. More recently, Pique et al. (2018) developed a novel method called oncomix to assess bimodal gene expression and using RNA-seq data from the Cancer Genome Atlas (Cancer Genome Atlas 2012), identified a new oncogene candidate, CBX2 for invasive breast carcinoma.

Bimodality in gene expression is an attractive phenomenon because it reflects the presence of a substructure in the data that would not typically be uncovered if the data was assumed to be normally-distributed. More broadly speaking, the presence of two modes or more in a gene expression distribution naturally indicates subpopulations in the data. A mixture model (McLachlan and Peel 2000) is a statistical method that models data distributions with a defined combination of unimodal distributions, and therefore, has been a popular modeling option for retaining normal distributions while investigating the presence of clustering in the data (Mar and McLachlan 2003; McLachlan et al. 2002; Scrucca et al. 2016).

## Discovering new regulators of phenotype through measures of gene expression variability

**Fig. 2:**
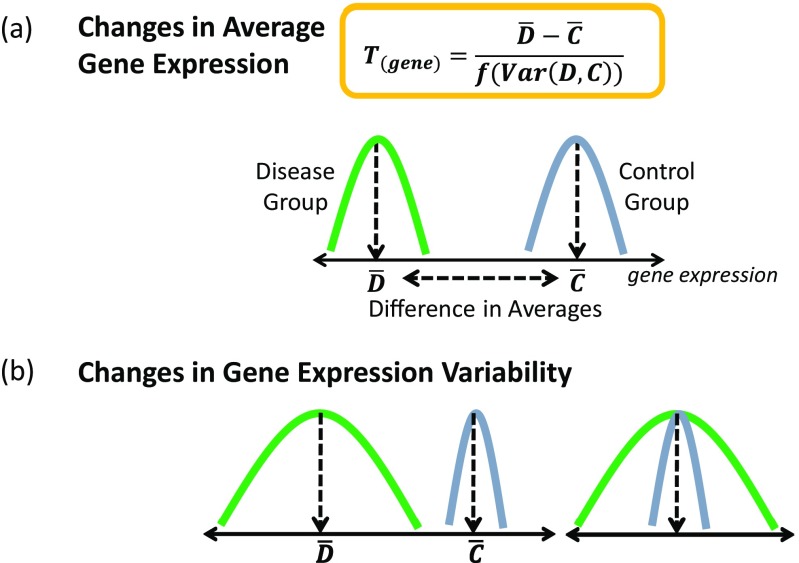
Contrasting differential average gene expression against differential variability in gene expression. **a** Differential expression relies upon identifying significant genes with a large difference in average expression and a small amount of variance. **b** Two scenarios are shown demonstrating how changes in variability of gene expression could occur between two phenotypic groups

There is increasing recognition that regulatory information can be derived from studying the variance of gene expression and not just the average effects which is the focus of differential expression (Fig. 2). Despite earlier studies (Ho et al. 2008), the overall uptake of the variance in the analysis of transcriptomic data has been slow to be incorporated. This may be because studying variance requires larger sample sizes as well as well-curated phenotypic data. Variance is a parameter that can be calculated from any statistical distribution and is certainly not exclusive to normality. However, the different degrees of variance observed when modeling gene expression levels suggest that fundamentally, the shape of the distribution is important and changeable with phenotype (Geiler-Samerotte et al. 2013).

A landmark study by Raj et al. (2010) on the nematode *Caenorhabditis elegans* (*C. elegans*) demonstrated how variable expression in a gene could determine the incomplete penetrance of a trait affecting the intestinal gut development. This study highlighted the regulatory impact of the inter-individual variability of gene expression because when one element of the gut development pathway was mutated, a downstream gene showed an increase in the variability of its gene expression. Consequently, this effect gave rise to a bimodal on/off expression of the downstream master regulator. Raj et al. observed that this variability was part of a thresholding effect where nematodes with a sufficiently high expression of the gene were able to activate downstream expression of the master regulator to ensure proper development of the intestinal gut. In another *C. elegans* study, Burga et al. (2011) showed that for a pair of synthetic lethal genes, tbx-8 and tbx-9, a mutation in either of these genes resulted in the increased inter-individual variability of gene expression in the other. Similarly, based on a thresholding effect, the expression of the synthetic lethal interactor was predictive of the phenotypic outcome.

In a study involving the human olfactory neurosphere-derived (hONS) stem cells, Mar et al. (2011) discovered that either direction of extreme change in the variability of gene expression could be associated with a disease phenotype. Specifically, Mar et al. observed a significant number of high-variability genes involved in stem cell regulation for hONS stem cells derived from patients with Parkinson’s disease. In contrast, a significant number of low-variability genes were observed for schizophrenia-derived hONS stem cells for the same stem cell pathways. Both disease groups were compared against a group of age and gender-matched control samples which also suggested that some degree of variability in gene expression is required for homeostasis. This was the first study to demonstrate that both increases and decreases in gene expression variability were a feature of human disease processes.

Using single-cell RNA-seq data collected from early-stage human embryos, Hasegawa et al. (2015) identified regulators of embryonic development using analyses based on inter-cellular variability of gene expression. Genes with the most stable inter-cellular expression variability over four developments stages, from a four-cell stage to blastocyst, were found to be enriched for those involving essentiality, haploinsufficiency, and ubiquitous expression. Hasegawa et al. also identified potential markers of stage based on changes in both variability and average expression, and found that *HDDC2,* a potential blastocyst marker validated experimentally in human embryonic stem cells and induced pluripotent stem cells.

## Jumping up (and down) to higher moments

In probability theory, a moment captures a specific property of the population distribution’s shape. Moments represent a powerful construct in statistics because they form the building blocks for the method of moments, a standard approach to estimating population parameters from data. Conceptually, the moments are sequential where the first moment equates to the mean or central location of the distribution. The second moment, the variance is a surrogate measure for how spread out the distribution is. Higher moments continue to add further layers of information about the shape of a distribution. It is worthwhile to note that in this context, the variance is just one of the multiple moments, and it follows that a natural extension may be to look to other moments to gain deeper insights into transcriptomic data.

**Fig. 3:**
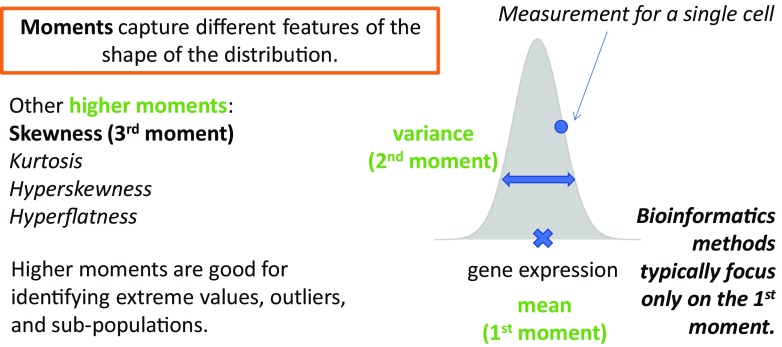
Moments characterize different properties of the distribution. The first three moments are shown using a hypothetical gene expression distribution collected from a population of single cells

Comprehensive investigations into studying higher moments from gene expression data are relatively few but are becoming increasingly relevant, especially with the discovery of new classes of non-coding RNAs which are generally expressed in only a minority of cells. For instance, skewness is the third moment which measures how disproportionate or unbalanced the data is distributed. A normal distribution has zero skewness, whereas highly skewed distributions are more prominently asymmetric. Casellas and Varona (2012) investigated the presence of skewness in four gene expression datasets using a flexible mixed model to account for asymmetry in the data. For cancer transcriptomes from the Cancer Genome Atlas, Marko and Weil (2012) studied the first four moments to determine the suitability of the normality assumption of four microarray datasets. Their results revealed that significant skewness and kurtosis were detected in the cancer gene expression datasets that they studied and as such, demonstrated that the data were not normally distributed (Fig. 3).

## Normally, genes interact with other genes

Although tests of differential gene expression assume independence between genes, we know that genes do, in fact, interact with other genes (Alon 2007). Identifying pairs of genes with significant co-expression patterns has become insightful for elucidating units of pathways and modules that may be co-regulated (Yeung et al. 2004). Metrics based on correlation have become a widely adopted strategy for inferring gene regulatory networks (Langfelder and Horvath 2008) or identifying differentially coordinated genes from gene expression data (Ghazanfar et al. 2018). As the focus shifts from a single gene to building relationships between multiple genes and their associated hierarchies, challenges arise on how to model these interactions appropriately. Issues such as non-linearity in gene expression and the need to account for a range of distributions call into question whether simple summary statistics or regression models that assume only a single distribution are adequate solutions.

## Single-cell sequencing is becoming the new (non)-normal for understanding cell biology

Advancements in next-generation sequencing methods have made the capture of individual transcriptional profiles from single cells feasible. Consequently, knowledge in every domain of biology is currently undergoing an explosive period of revision. Single-cell sequencing experiments are uncovering insights that their bulk sample counterparts had previously missed, and innovative discoveries are adding new depth to how we understand the genome. Given the unprecedented degree of heterogeneity in single-cell sequencing data, it is, therefore, not surprising that big data analysis represents the main gateway to these discoveries at this time. Unlike ensemble-level transcriptomes (Levsky and Singer 2003), observations that gene expression profiles from single cells are non-normal have been made as early as 2005 (Bengtsson et al. 2005) with independent validation provided from more accurate and modern technologies (Leng et al. 2013; Moignard et al. 2015). Multimodal distributions are a key feature of single-cell gene expression data (Shalek et al. 2013), both due to the increased prevalence of zeros from technical drop-out, and the representation of new subpopulations or subtypes in the cell population. For a broad range of applications, spanning pre-processing to regulatory network inference, statistical methods that are based on mixture models or hierarchical models have been employed to account for the multi-modality in this data (Chen and Mar 2018).

## Future directions

The growth of transcriptomic data continues to march forward with an expansion into multiple directions covering technology, volume, complexity, and type. How will our set of statistical methods adapt and evolve to meet the next generation of big data analysis challenges? While the examples in this review have focused on gene expression data, issues of non-normality are pertinent to other kinds of “omic” data too. Cell-free-based assays are now routinely used in clinical settings where genomic and epigenomic datasets are collected for applications such as tumor profiling (Adalsteinsson et al. 2017) and prenatal testing (Yin et al. 2018). These datasets involve a heterogeneous mix of cells that stem from multiple sources, either tumor versus normal, or mother versus embryo, and hence, decomposing the data using non-normal distributions are necessary for accurate biological inferences to be made. Similarly, increasing evidence points to genetic mosaicism as a more widespread phenomenon than previously thought, with studies suggesting that this may be a normative process affecting all human beings (Campbell et al. 2015). Consequently, modeling genes with mixtures of distributions to account for the mosaicism could be one approach to be adopted. These two examples could seamlessly be substituted for many others, but the recurring theme of non-normality endures. As transcriptomics becomes more specialized and personalized, it remains impossible to know for sure how the analysis of big data will change in the future. Nevertheless, the next wave of big data research is set to be anything but normal.
